# Genome Sequence of the Urethral Isolate *Pseudomonas aeruginosa* RN21

**DOI:** 10.1128/genomeA.00788-15

**Published:** 2015-07-16

**Authors:** Daniel Wibberg, Petra Tielen, Maike Narten, Max Schobert, Jochen Blom, Sarah Schatschneider, Ann-Kathrin Meyer, Rüdiger Neubauer, Andreas Albersmeier, Stefan Albaum, Martina Jahn, Alexander Goesmann, Frank-Jörg Vorhölter, Alfred Pühler, Dieter Jahn

**Affiliations:** a Center for Biotechnology, CeBiTec, Universität Bielefeld, Bielefeld, Germany; b Institute of Microbiology, Technische Universität, Braunschweig, Germany; c Urological Clinic, Kassel, Germany

## Abstract

*Pseudomonas aeruginosa* is known to cause complicated urinary tract infections (UTI). The improved 7.0-Mb draft genome sequence of *P. aeruginosa* RN21, isolated from a patient with an acute UTI, was determined. It carries three (pro)phage genomes, genes for two restriction/modification systems, and a clustered regularly interspaced short palindromic repeats (CRISPR)/CRISPR-associated (Cas) system.

## GENOME ANNOUNCEMENT

*Pseudomonas aeruginosa* is one of most frequently occurring Gram-negative nosocomial pathogens and is known as one of the major agents causing complicated urinary tract infections (UTIs) (1, 2). These infections are predominantly biofilm-related phenomena, influenced by the action of lytic bacteriophages (3, 4). Here, we report the draft genome sequence of *P. aeruginosa* RN21 isolated from a patient with an acute UTI (5). The strain was urease negative and had a moderately virulent phenotype without remarkable antibiotic resistances. In order to obtain the draft genome sequence, we extracted genomic DNA from this isolate to construct a paired-end library for shotgun sequencing on a genome sequencer FLX (GS FLX) system using Titanium technology (Roche) as described recently (6, 7, 8). Standard protocols were applied per the manufacturer’s instructions. The sequencing run yielded 216,518,852 bases from 958,125 aligned individual reads, among them 345,167 paired-end reads. The assembly obtained by applying the GS Assembly software resulted in 216 contigs (>500 bp), of which 160 were organized into 9 scaffolds. An *in silico* gap closure approach (9, 10) was performed, which reduced the number of contigs to 83. The improved draft genome had a total size of 6,970,506 bp with an average coverage of 31.1×. The G+C content of the genome was 65.88%. Automated genome annotation was carried out by means of the GenDB platform (11). This resulted in the prediction of 6,422 protein-coding sequences (CDSs). Three copies of the rRNA operons and 60 tRNAs were predicted. Identification of RN21 singletons in comparison to the *P. aeruginosa* core genome was established using the platform EDGAR (12). Most of the unique genes were related to three (pro)phage genomes and to multiple protection systems against phages and other foreign DNA. In detail, a complete D3112-like virus genome (PARN21_0994 to PARN21_1037), a novel phage genome containing multiple genes with strong homology to phage D3 genes (PARN21_3655 to PARN21_3727), and a leukocidin-like cytotoxin gene containing (pro)phages (PARN21_6249 to PARN21_6290) were found (13, 14). A genomic island (PARN21_2458 to PARN21_2502) encoded type II and III restriction/modification systems, a glutathione *S*-transferase, and a filamentous hemagglutinin-like protein with corresponding labile enterotoxin output protein (15, 16). Similarly, the unique genomic region PARN21_2633 to PARN21_2669 encoded various DNA methylases, endonucleases, RecT, LexA-type regulators, an FlsK homologue, and structural maintenance of chromosomes (SMC) domain proteins, possibly involved in chromosome assembly (17). These observations were completed by the detection of a set of genes encoding the proteins recognizing clustered regularly interspaced short palindromic repeats (CRISPR), a complete CRISPR-associated (Cas) system (PARN21_4342 to PARN21_4350) (18). Finally, CDSs for lipopolysaccharide (LPS)-modifying enzymes (PARN21_1897 to PARN21_1899 and PARN21_2688), a capsule biosynthesis enzyme (PARN21_4510), a type IV secretion system (PARN21_6231 to PARN21_6236), an antitoxin (PARN21_5028/59), and multiple potential transcriptional regulators were found (19–21). A more detailed and comparative analysis of this genome will contribute to a deeper understanding of urinary tract infections caused by *P. aeruginosa* and may advance our conception of its phage interactions.

### Nucleotide sequence accession numbers. 

This whole-genome shotgun project has been deposited in DDBJ/EMBL/GenBank under the accession numbers CGFY01000001 to CGFY01000083. The version described in this paper is the first version, CGFY01000000.1.

## References

[1] Mittal R, Aggarwal S, Sharma S, Chhibber S, Harjai K 2009 Urinary tract infections caused by *Pseudomonas aeruginosa*: a minireview. J Infect Public Health 2:101–111. doi:10.1016/j.jiph.2009.08.003. 20701869

[2] Ronald A 2003 The etiology of urinary tract infection: traditional and emerging pathogens. Dis Mon 49:71–82. doi:10.1067/mda.2003.8. 12601338

[3] Laverty G, Gorman SP, Gilmore BF 2014 Biomolecular mechanisms of *Pseudomonas aeruginosa* and *Escherichia coli* biofilm formation. Pathogens 3:596–632. doi:10.3390/pathogens3030596. 25438014 PMC4243431

[4] Ahiwale S, Tamboli N, Thorat K, Kulkarni R, Ackermann H, Kapadnis B 2011 *In vitro* management of hospital *Pseudomonas aeruginosa* biofilm using indigenous T7-like lytic phage. Curr Microbiol 62:335–340. doi:10.1007/s00284-010-9710-6. 20711581

[5] Tielen P, Narten M, Rosin N, Biegler I, Haddad I, Hogardt M, Neubauer R, Schobert M, Wiehlmann L, Jahn D 2011 Genotypic and phenotypic characterization of *Pseudomonas aeruginosa* isolates from urinary tract infections. Int J Med Microbiol 301:282–292. doi:10.1016/j.ijmm.2010.10.005. 21193347

[6] Vorhölter FJ, Tielen P, Wibberg D, Narten M, Schobert M, Tüpker R, Blom J, Schatschneider S, Winkler A, Albersmeier A, Goesmann A, Pühler A, Jahn D 2015 Genome sequence of the urethral catheter isolate *Pseudomonas aeruginosa* MH19. Genome Announc 3(2):e00115-15. doi:10.1128/genomeA.00115-15. PMC4357764 25767242

[7] Köhler KA, Rückert C, Schatschneider S, Vorhölter FJ, Szczepanowski R, Blank LM, Niehaus K, Goesmann A, Pühler A, Kalinowski J, Schmid A 2013 Complete genome sequence of *Pseudomonas* sp. strain VLB120 a solvent tolerant, styrene degrading bacterium, isolated from forest soil. J Biotechnol 168:729–730. doi:10.1016/j.jbiotec.2013.10.016. 24161918

[8] Schwientek P, Szczepanowski R, Rückert C, Kalinowski J, Klein A, Selber K, Wehmeier UF, Stoye J, Pühler A 2012 The complete genome sequence of the acarbose producer *Actinoplanes* sp. SE50/110. BMC Genomics 13:112. doi:10.1186/1471-2164-13-112. 22443545 PMC3364876

[9] Maus I, Wibberg D, Stantscheff R, Cibis K, Eikmeyer FG, König H, Pühler A, Schlüter A 2013 Complete genome sequence of the hydrogenotrophic archaeon *Methanobacterium* sp. Mb1 isolated from a production-scale biogas plant. J Biotechnol 168:734–736. doi:10.1016/j.jbiotec.2013.10.013. 24184088

[10] Wibberg D, Blom J, Jaenicke S, Kollin F, Rupp O, Scharf B, Schneiker-Bekel S, Sczcepanowski R, Goesmann A, Setubal JC, Schmitt R, Pühler A, Schlüter A 2011 Complete genome sequencing of *Agrobacterium* sp. H13-3, the former *Rhizobium lupini* H13-3, reveals a tripartite genome consisting of a circular and a linear chromosome and an accessory plasmid but lacking a tumor-inducing Ti-plasmid. J Biotechnol 155:50–62. doi:10.1016/j.jbiotec.2011.01.010. 21329740

[11] Meyer F, Goesmann A, McHardy AC, Bartels D, Bekel T, Clausen J, Kalinowski J, Linke B, Rupp O, Giegerich R, Pühler A 2003 GenDB—an open source genome annotation system for prokaryote genomes. Nucleic Acids Res 31:2187–2195. doi:10.1093/nar/gkg312. 12682369 PMC153740

[12] Blom J, Albaum SP, Doppmeier D, Pühler A, Vorhölter FJ, Zakrzewski M, Goesmann A 2009 EDGAR: a software framework for the comparative analysis of prokaryotic genomes. BMC Bioinformatics 10:154. doi:10.1186/1471-2105-10-154. 19457249 PMC2696450

[13] Heo YJ, Chung I-Y, Choi KB, Lau GW, Cho YH 2007 Genome sequence comparison and superinfection between two related *Pseudomonas aeruginosa* phages, D3112 and MP22. Microbiology 153:2885–2895. doi:10.1099/mic.0.2007/007260-0. 17768233

[14] Homma JY, Matsuura M, Shibata M, Kazuyama Y, Yamamoto M, Kubota Y, Hirayama T, Kato I 1984 Production of leukocidin by clinical isolates of *Pseudomonas aeruginosa* and antileukocidin antibody from sera of patients with diffuse panbronchiolitis. J Clin Microbiol 20:855–859. 6439729 10.1128/jcm.20.5.855-859.1984 PMC271458

[15] Wyszomirski KH, Curth U, Alves J, Mackeldanz P, Möncke-Buchner E, Schutkowski M, Krüger DH, Reuter M 2012 Type III restriction endonuclease EcoP15I is a heterotrimeric complex containing one res subunit with several DNA-binding regions and ATPase activity. Nucleic Acids Res 40:3610–3622. doi:10.1093/nar/gkr1239. 22199260 PMC3333885

[16] Clantin B, Hodak H, Willery E, Locht C, Jacob-Dubuisson F, Villeret V 2004 The crystal structure of filamentous hemagglutinin secretion domain and its implications for the two-partner secretion pathway. Proc Natl Acad Sci U S A 101:6194–6199. doi:10.1073/pnas.0400291101. 15079085 PMC395945

[17] Song D, Loparo JJ 2015 Building bridges within the bacterial chromosome. Trends Genet 31:164–173. doi:10.1016/j.tig.2015.01.003. 25682183

[18] Barrangou R 2015 The roles of CRISPR-Cas systems in adaptive immunity and beyond. Curr Opin Immunol 32:36–41. doi:10.1016/j.coi.2014.12.008. 25574773

[19] Bélanger M, Burrows LL, Lam JS 1999 Functional analysis of genes responsible for the synthesis of the B-band O antigen of *Pseudomonas aeruginosa* serotype O6 lipopolysaccharide. Microbiology 145:3505–3521. 10627048 10.1099/00221287-145-12-3505

[20] Finke A, Roberts I, Boulnois G, Pzzani C, Jann K 1989 Activity of CMP-2-keto-3-deoxyoctulosonic acid synthetase in *Escherichia coli* strains expressing the capsular K5 polysaccharide implication for K5 polysaccharide biosynthesis. J Bacteriol 171:3074–3079. 2542215 10.1128/jb.171.6.3074-3079.1989 PMC210017

[21] Wallden K, Rivera-Calzada A, Waksman G 2010 Type IV secretion systems: versatility and diversity in function. Cell Microbiol 12:1203–1212. doi:10.1111/j.1462-5822.2010.01499.x. 20642798 PMC3070162

